# Supplementary material for: Benchmarking long-read genome sequence alignment tools for human genomics applications
Source: PeerJ. 2023 Dec 18;11:e16515. doi: 10.7717/peerj.16515 (PMC10734412; doi:10.7717/peerj.16515)

S5: Nanopore SVs 10,001 – 100,000 bp

Deletions

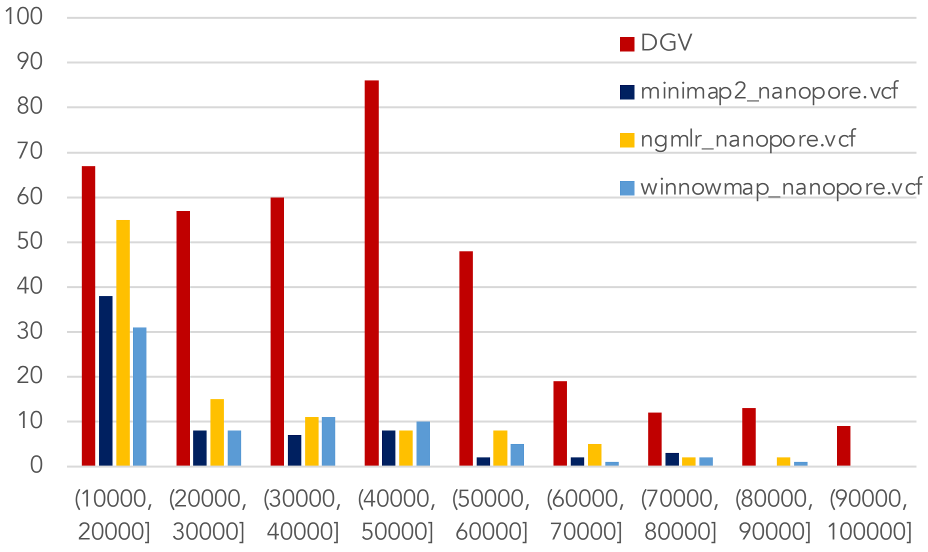

Insertions

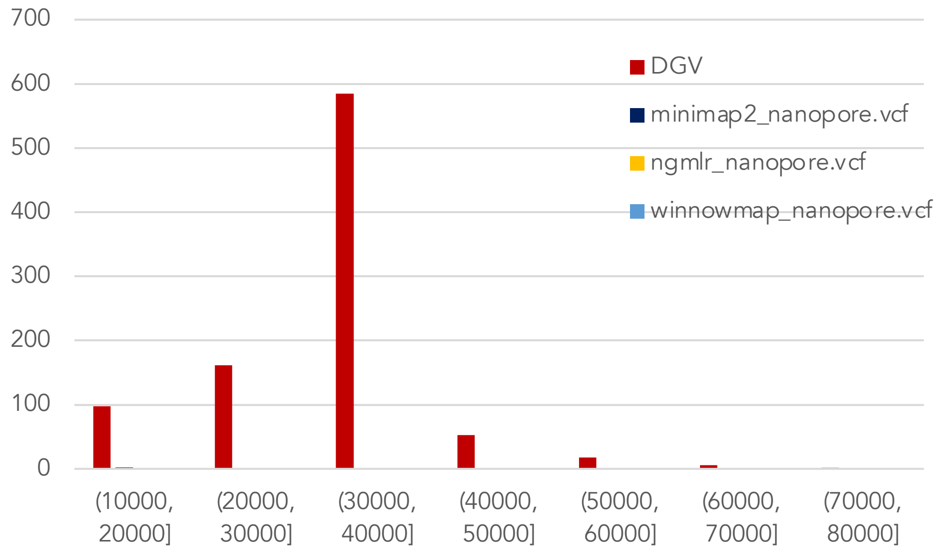

Inversions

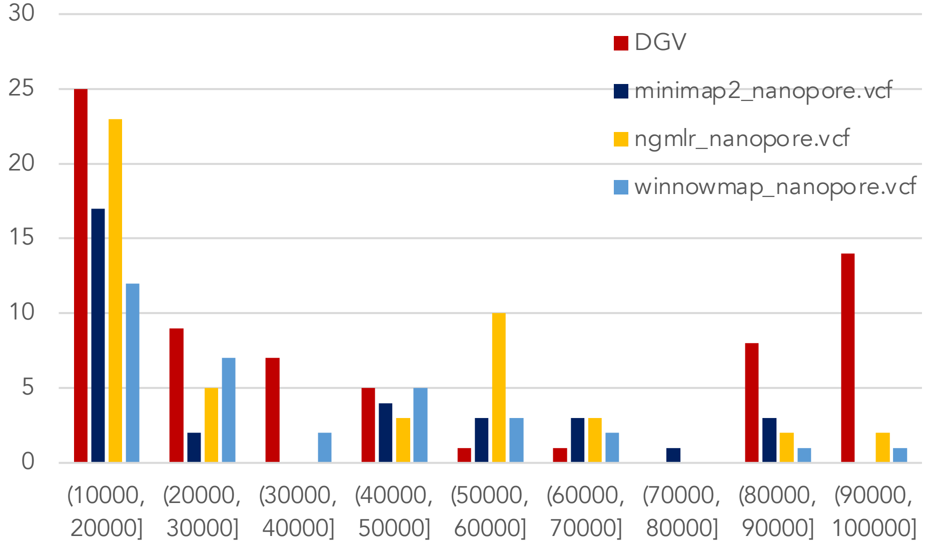

Duplications

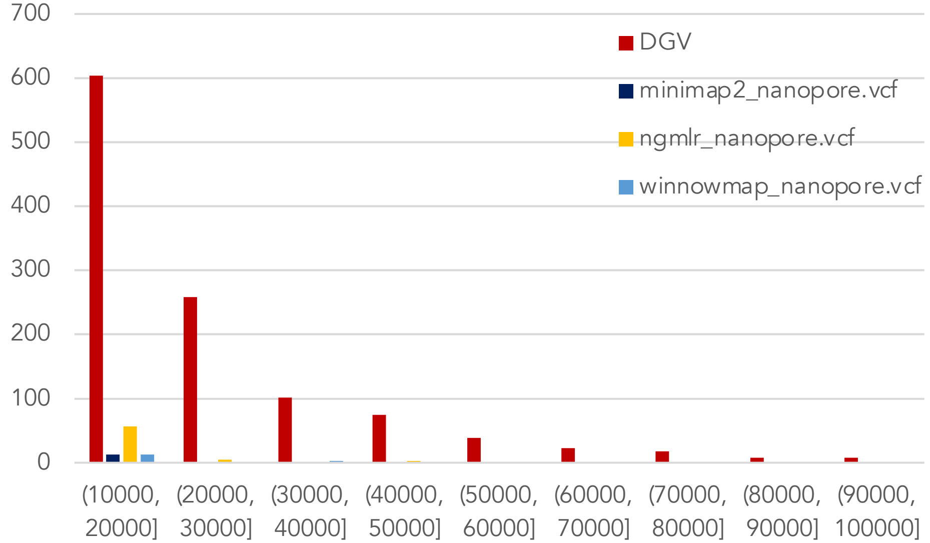

S6: SMRT-CCS SVs 10,001 – 100,000 bp

Deletions

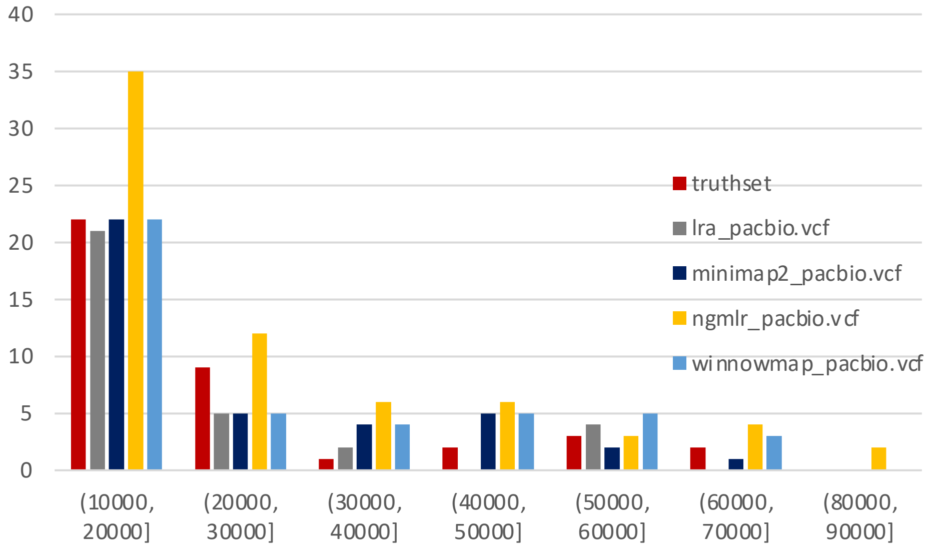

Insertions

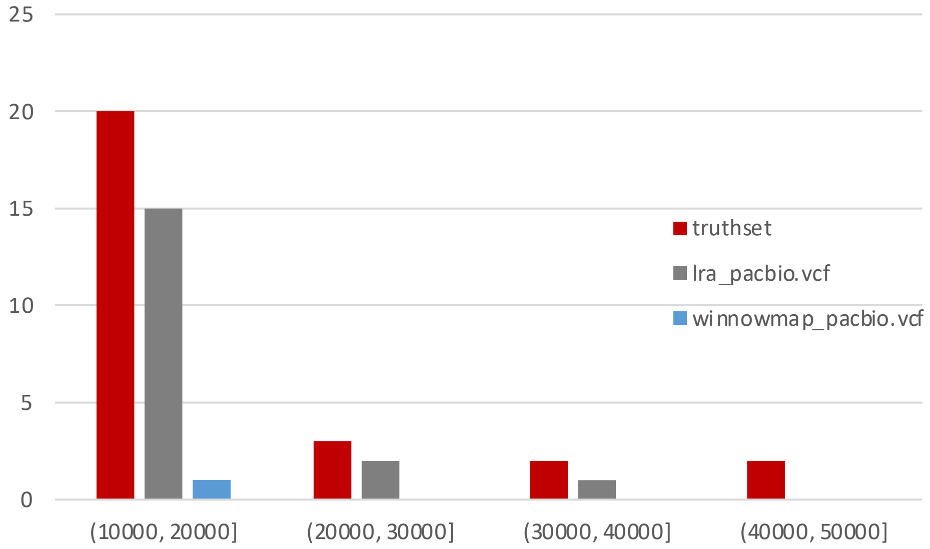

Duplications

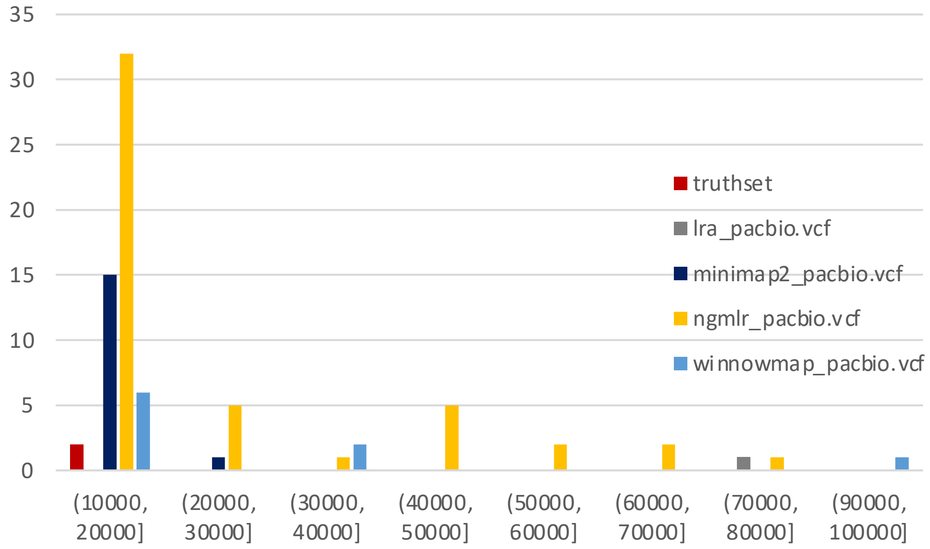

Supplement: Supplemental Information 5 — These figures represent the largest structural variants present in callsets from manuscript analyses. [file peerj-11-16515-s005.pdf]
